# A76 PHYSICIANS PERCEIVED VALUE OF COMPUTED TOMOGRAPHY SCANS FOR INFLAMMATORY BOWEL DISEASE PATIENTS IN THE EMERGENCY DEPARTMENT: A SURVEY OF CANADIAN PHYSICIANS

**DOI:** 10.1093/jcag/gwac036.076

**Published:** 2023-03-07

**Authors:** C Roda, I Stiell, C Dube, B Macdonald, H Moloo, A DeBuck, J McCurdy

**Affiliations:** 1 Internal Medicine; 2 Emergency Medicine; 3 Gastroenterology; 4 Radiology; 5 Surgery, The Ottawa Hospital , Ottawa; 6 Surgery, Mount Sinai Hospital, Toronto, Canada

## Abstract

**Background:**

Despite concerns regarding the risks of ionizing radiation from computed tomography (CT) imaging, the rates of abdominal pelvic CT (APCT) utilization in the emergency department (ED) continues to increase for patients with inflammatory bowel disease (IBD).

**Purpose:**

To determine the factors that drive decisions on when to perform APCT imaging in the ED for patients with IBD and to determine if differences exist between physician specialties.

**Method:**

We performed a quantitative, web-based survey between November 2021 and August 2022. Structured questions for Crohn’s disease (CD) and ulcerative colitis (UC) were developed with input from stakeholders in gastroenterology, surgery, and emergency medicine. Surveys were disseminated to Canadian physicians in each of the three specialities through personal emails, and by newsletter from national specialty organizations. Between specialty comparisons were performed by Chi-squared and Fisher exact tests where appropriate.

**Result(s):**

A total of 208 participants responded to our survey: median age 44 years (IQR 37-50), 132 (63%) male, and 141 (68%) in an academic practice. Survey participants included 81 (39%) gastroenterologists, 35 (17%) surgeons and 92 (44%) emergency physicians.

There were significant differences between specialties in the perceived rates of positive findings from APCT imaging. In UC, gastroenterologists felt inflammation alone was more common than emergency physicians and bowel obstruction and septic complications less common than surgeons and emergency physicians. In CD, surgeons felt bowel obstructions, septic complications and bowel perforations were more common compared with gastroenterologists and emergency physicians.

There were significant differences between specialties in the types of clinical presentations that drove decisions to arrange APCT imaging (Figure 1a&b). In UC, gastroenterologists were less likely to order APCT imaging for diarrhea with rectal bleeding, abdominal pain without peritoneal findings and fever than surgeons and emergency physicians. In CD, there were similar practice patterns between specialities except gastroenterologists were less likely to order APCT imaging for diarrhea with rectal bleeding than surgeons.

**Image:**

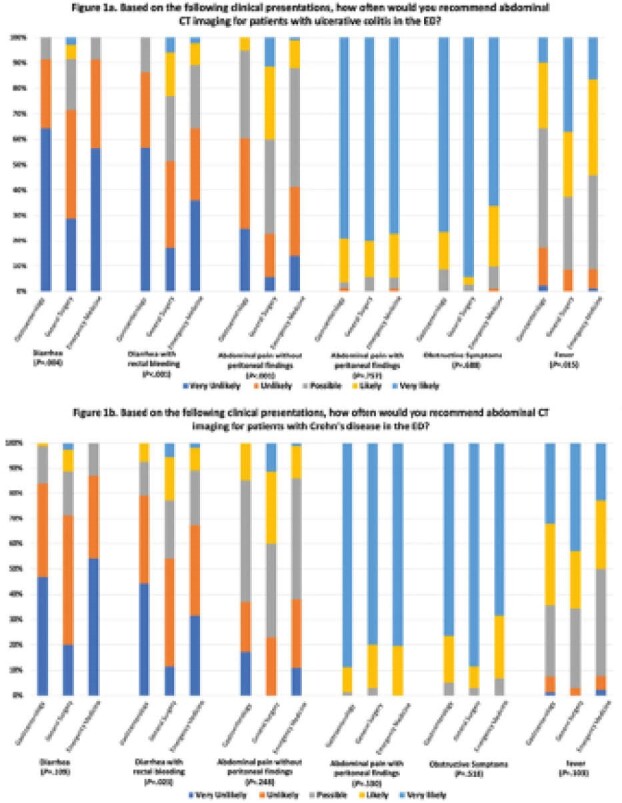

**Conclusion(s):**

Our survey identified key differences between physician specialties in the perceived rates of positive findings from APCT imaging and practice patterns of CT utilization. These findings will help to guide the development of future multidisciplinary consensus guidelines for the appropriateness of CT imaging in the ED for patients with IBD.

**Please acknowledge all funding agencies by checking the applicable boxes below:**

None

**Disclosure of Interest:**

None Declared

